# Pelvic actinomycosis three years after colporrhaphy: A case report

**DOI:** 10.1016/j.crwh.2021.e00359

**Published:** 2021-09-21

**Authors:** Stephen Davick, Samiksha Annira, Tyler Schwiesow

**Affiliations:** a University of Iowa – Des Moines Internal Medicine Residency Program, 1415 Woodland Ave. Ste. 140, Des Moines, IA 50309, United States; b University of Iowa Hospitals & Clinics, 200 Hawkins Drive, Iowa City, IA 52242, United States

**Keywords:** Actinomyces, Pelvic actinomycosis, Prolapse, Pelvic reconstructive surgery, Colporrhaphy

## Abstract

A 57-year-old patient presented with vaginal discharge and was found to have a pelvic abscess with *A. turicensis* and *Streptococcus constellatus*, with likely nidus of infection being a non-absorbable suture placed during colporrhaphy three years prior. She was treated with drain placement and antibiotics. Post-hospitalization, her colporrhaphy suture was removed. Subsequently the drain output decreased and this was removed as well. She had a total course of 6 weeks of amoxicillin/clavulanate, with complete resolution of her abscess.

## Highlights

• *Actinomyces* pelvic abscess can occur after colporrhaphy with non-absorbable suture.
• Patients with pelvic abscess may have actinomycosis even if they do not have an intrauterine device.
• Removal of the source of infection is important in the treatment of pelvic actinomycosis.

## 1 Introduction

Actinomycosis is a rare, invasive infection caused by bacteria from the *Actinomyces* family. These organisms are gram-positive, pigment-producing, non-spore-forming bacteria that form branching filaments.

*Actinomyces* normally colonize the oropharyngeal, gastrointestinal, and female genital tracts as a commensal organism [1]. *Actinomyces* infection often results from disruption to the mucosal barrier within these tracts and is most often seen in patients with poor dental hygiene, post-dental procedures, post-abdominal and pelvic surgeries, and in patients with intrauterine devices (IUDs) [2]. In gynecology, case reports on actinomycosis remain rare and are most often associated with IUDs [3,4]. In this report, a case is described of pelvic actinomycosis associated with the surgical sutures from a colporrhaphy which was performed for pelvic organ prolapse three years prior to the patient's hospitalization.

## 2 Case presentation

A 57-year-old multiparous woman with a history of cystocele which was corrected with colporrhaphy three years prior presented to the hospital with four weeks of sharp, unrelenting right-sided gluteal pain that radiated down her right leg; the pain was worse with urination and defecation and associated with red-brown and purulent vaginal discharge. Medical history was notable for oral hormone replacement with estradiol, otherwise not relevant to her presenting condition. Surgical history was notable for colporrhaphy three years prior, as well as abdominal hysterectomy eighteen years prior for ovarian cysts. She smoked a half-pack of cigarettes per day and was monogamous with her husband, and neither of them had ever had a sexually transmitted infection.

**Fig. 1:**
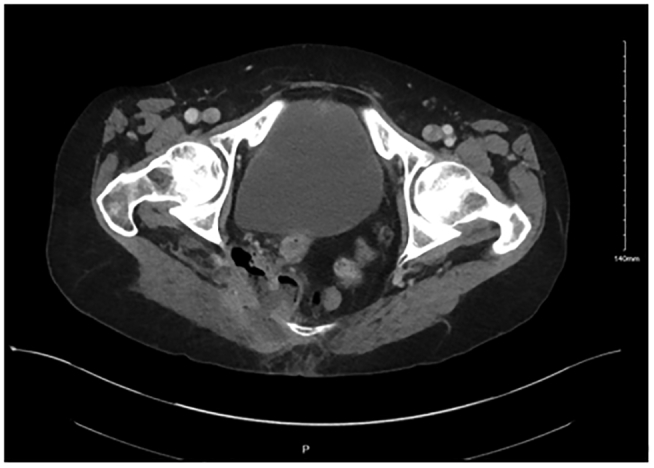
Pelvic abscess on hospital day 0 CT scan.

Upon initial evaluation, vital signs were notable for temperature of 37.9 degrees Celsius and a blood pressure of 96/55 mmHg. Serologies demonstrated hypokalemia of 2.8 mmol/L, white blood cell count of 14,980/uL, and were otherwise unremarkable. Urinalysis was cloudy with trace protein, red blood cells 26–50/hpf, white blood cells >50/hpf, bacteria >50/hpf, and epithelial cells >10/hpf; culture grew mixed urogenital flora all at <10,000 cfu/mL. Vaginitis screen was negative for trichomonas, *Gardnerella vaginalis*, and *Candida* species. Computerized tomography (CT) of the abdomen and pelvis with contrast showed a 6.0 × 5.0 × 5.9 cm multiloculated abscess along the right lateral pelvic wall and within the gluteus maximus (Fig. 1).

She was admitted to the hospital, started on ceftriaxone and metronidazole, and gynecology and interventional radiology were consulted. A drain was placed into the abscess by the interventional radiologist on hospital day 1. The gynecology consultant considered the patient's nidus for infection to be the non-absorbable sutures from her colporrhaphy. Cultures from the wound demonstrated heavy growth of *A. turicensis* and moderate growth of *Streptococcus constellatus* on hospital day 4. Antibiotics were then transitioned to oral amoxicillin/clavulanate 875/125 mg twice daily. She was discharged home on hospital day 4 in good condition, on antibiotics and with her drain in place.

**Fig. 2:**
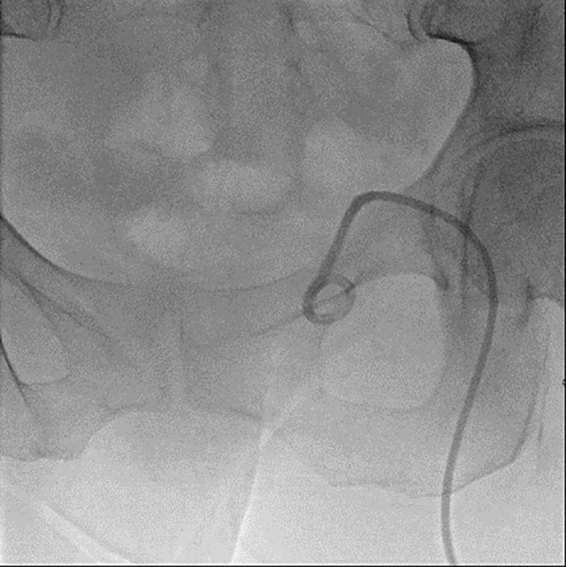
Interventional radiologist-guided injection of contrast into abscess space showing resolution of abscess 50 days after initial presentation.

Six days post-discharge, the interventional radiologist replaced the patient's drain to access a deeper area of the abscess. Eleven days post-discharge, she had one stitch removed at the bedside during follow-up with gynecology, who also confirmed both by chart review and discussion with the surgeon who performed the colporrhaphy that there was a second suture placed in the right apex of the vagina and tied down to the right uterosacral ligament. On follow-up with infectious disease, she was continued on her antibiotic course for a total of 6 weeks, after which they determined her abscess had resolved. Her drain was removed by the interventional radiologist 50 days post-hospitalization after fluoroscopic evaluation demonstrated complete drainage of the cavity (Fig. 2). She then underwent surgical exploration 53 days post-discharge, and no additional sutures or foreign bodies were identified. During post-surgical follow-up with gynecology, they determined that her abscess had completely resolved.

## 3 Discussion

*Actinomyces* is a commensal organism within the oral cavity, gastrointestinal and genital tracts and typically is not pathogenic in nature. However, in the event of trauma or surgery or other insult that compromises the integrity of mucosal tissue, *Actinomyces* is capable of causing invasive disease [5]. Pelvic actinomycosis is typically insidious and can go undetected for months to years before ultimately presenting as nonspecific symptoms, including low-grade fever, lower abdominal pain, and weight loss [6]. Among gynecologic patients, pelvic actinomycosis typically occurs secondary to IUD use [7]. In this patient, the non-absorbable sutures from colporrhaphy served as the nidus for an *Actinomyces* infection to develop. Colporrhaphy procedures have been phased out in recent years as techniques in pelvic floor reconstruction surgery have evolved to incorporate the use of synthetic mesh for pelvic organ prolapse [8]. In the literature, mesh-related complications typically arise from infection with vaginal flora, including *Actinomyces* in very rare instances [9]. Interestingly, there are few reports that document the incidence of pelvic actinomycosis resulting from pelvic reconstructive procedures [[10], [11], [12], [13], [14]]. Although both are foreign bodies, actinomycosis associated with non-absorbable suture is much less known than actinomycosis associated with IUDs. A MEDLINE search for the terms “colporrhaphy” and “*Actinomyces*” from January 1950 to August 2021 in all languages producesminimal documented evidence of *Actinomyces* infection among patients who have undergone colporrhaphy. Further investigation of the infection rate between colporrhaphy and vaginal mesh procedures may provide insight into whether there is a preferable technique to minimize infection risk [15,16].

## 4 Conclusion

This patient demonstrated a unique presentation of *Actinomyces* infection. Though pelvic *Actinomyces* infections are most often associated with IUD usage, this case exhibits the possibility for invasive disease to arise from pelvic reconstructive surgery, which is known to occur in rare instances as described by the literature. This patient highlighted the importance of early consideration of the possibility of *Actinomyces* infection in the setting of a pelvic abscess in a gynecologic patient.
